# Association of *MSH2* Expression With Tumor Mutational Burden and the Immune Microenvironment in Lung Adenocarcinoma

**DOI:** 10.3389/fonc.2020.00168

**Published:** 2020-02-21

**Authors:** Mingming Jia, Linli Yao, Qin Yang, Tian Chi

**Affiliations:** ^1^School of Life Science and Technology, ShanghaiTech University, Shanghai, China; ^2^CAS Center for Excellence in Molecular Cell Science, Shanghai Institute of Biochemistry and Cell Biology, Chinese Academy of Sciences, Shanghai, China; ^3^University of Chinese Academy of Sciences, Beijing, China; ^4^State Key Laboratory of Oncogenes and Related Genes, Shanghai Cancer Institute, Ren Ji Hospital, School of Medicine, Shanghai Jiao Tong University, Shanghai, China; ^5^Department Immunobiology, Yale University School of Medicine, New Haven, CT, United States

**Keywords:** lung adenocarcinoma, *MSH2* expression, biomarkers, immunotherapy, tumor mutation burden

## Abstract

Immune checkpoint blockade (ICB) therapies that target programmed cell death 1 (PD1) and PD1 ligand 1 (PDL1) have demonstrated promising benefits in lung adenocarcinoma (LUAD), and tumor mutational burden (TMB) is the most robust biomarker associated with the efficacy of PD-1-PD-L1 axis blockade in LUAD, but the assessment of TMB by whole-exome sequencing (WES) is rather expensive and time-consuming. Although targeted panel sequencing has been developed and approved by the US Food and Drug Administration (FDA) to estimate TMB, we found that its predictive accuracy for ICB response was significantly lower than WES in LUAD. Given that previous studies were mainly focusing on genomic variations to explore surrogate biomarkers of TMB, we turned to examine the transcriptome-based correlation with TMB in this study. Combining three immunotherapeutic cohorts with two independent The Cancer Genome Atlas (TCGA) datasets, we revealed that the expression of mutS homolog 2 (*MSH2*), one of the most crucial genes involved in DNA mismatch repair (MMR) pathway, was the strongest feature associated with increased TMB in multivariate analysis. Furthermore, *MSH2* expression also displayed a significantly positive correlation with smoking signature while an inverse association with MMR deficiency (MMRd) signature in LUAD. More importantly, high expression of *MSH2* markedly correlated with increased *PD-L1* expression and CD8+ T cell infiltration, both suggesting a prominent immunotherapy-responsive microenvironment in LUAD. Notably, detecting *MSH2* expression is much easier, faster, and cheaper than TMB in clinical practice. Taken together, this study demonstrates the association of *MSH2* expression with TMB and the immune microenvironment in LUAD. *MSH2* expression may be developed as a potential surrogate biomarker of TMB to identify ICB responders in LUAD.

## Introduction

Recent clinical trials with immune checkpoint blockade (ICB) therapies have demonstrated durable clinical responses in patients with non-small cell lung cancer (NSCLC), but only a minority of patients respond (1–3). The combination of ICB therapies can improve response rates but also result in more severe adverse effects than single-agent therapy (4). Previous studies have reported that tumor mutational burden (TMB) (1–3, 5–7), programmed death-ligand 1 (PD-L1) expression (5, 8), CD8+ T cell infiltration (1, 9, 10), and DNA mismatch repair deficiency (MMRd) (5, 11) could affect the efficacy of PD-1 blockade immunotherapy (5, 12, 13). However, only TMB and PD-L1 expression are validated as predictive biomarkers for ICB response in phase III clinical trials across multiple cancer types (5, 7).

Currently, TMB performs much better than other biomarkers for predicting ICB response in NSCLC (1–3). High TMB can potentially generate higher immunogenic neoantigens presented on the tumor cell surface and then facilitate immune recognition of tumor cells as foreign (1, 2, 7). However, the assessment of TMB is expensive and time-consuming (6, 14). Alternatively, PD-L1 expression assessed by immunohistochemistry (IHC) is much cheaper and timelier to select candidates for ICB therapies, but many patients whose tumors are PD-L1-positive do not respond (1). Additionally, the localization (on tumor-infiltrating immune cells or tumor cells) and positivity threshold of PD-L1 expression for predicting ICB efficacy are still undetermined, which may affect its clinical application (1, 5, 6, 8, 15). Therefore, we hypothesized that other factors, which highly correlated with increased TMB and were as convenient as PD-L1 expression to be detected, might also be developed as potential biomarkers to predict ICB response in NSCLC.

To test our hypothesis, we recruited three well-studied NSCLC immunotherapeutic cohorts (1–3) and one multidimensional non-immunotherapeutic The Cancer Genome Atlas (TCGA) dataset. As previous studies reported (2, 10, 16–19), TCGA samples without ICB therapies are still informative to explore tumor immune escape and can also derive surrogate biomarkers for ICB therapies. Combining these four cohorts, we revealed that *MSH2* expression was the most robust feature associated with increased TMB and smoking signature in multivariate analysis and might be developed as a potential surrogate biomarker of TMB for identifying ICB responders in lung adenocarcinoma (LUAD), one of the commonest types of NSCLC (20, 21).

## Materials and Methods

### Clinical Immunotherapeutic Patients

Given the intratumoral heterogeneity across different cancer subtypes, it is more reliable to discover the specific determinants for ICB efficacy within the same cancer subtype (5), so we only focused on the LUAD subtype according to its dominating proportion in previous NSCLC immunotherapeutic cohorts (1–3). We collected three LUAD cohorts containing both clinical and genomic characteristics, which were initially reported in *Science* (1), *Journal of Clinical Oncology (JCO)* (3), and *Cancer Cell* (2) journals. For the *Science-LUAD* cohort, it contained 29 LUAD patients treated with PD-1 blockade (pembrolizumab) (1). For the *Cancer Cell-LUAD* cohort, it involved 59 LUAD patients treated with PD-1 plus CTLA-4 blockade (nivolumab plus ipilimumab) (2). For the *JCO-LUAD* cohort, it contained 186 LUAD patients who had received anti-PD-(L)1 monotherapy or in combination with anti-CTLA-4 (3).

### *TCGA-LUAD* Datasets Without Immunotherapy

Non-immunotherapeutic *TCGA-LUAD* datasets were extracted from the UCSC Xena multi-omics database platform (22) (https://tcga.xenahubs.net), including somatic mutation (*n* = 543) and RNA-seq expression (*n* = 576) profiles. We first removed adjacent normal samples from RNA-seq expression data and then only analyzed those LUAD samples that had both genomic and transcriptomic profiles (*n* = 478).

### Tumor Mutational Burden (TMB) Estimates

TMB was defined as the number of somatic non-synonymous single nucleotide variants. Raw somatic mutation data in three immunotherapeutic cohorts were extracted from the respective Supplementary Materials (1–3). Mutation profiles were assessed by whole-exome sequencing (WES) on the Illumina platform in *Science-LUAD* (1) and *Cancer Cell-LUAD* (2) cohorts while determined by MSK-IMPACT targeted panel sequencing on specific cancer-associated genes in *JCO-LUAD* (3) cohort. The detailed methodology for generating mutation calls has previously been described (1–3). For the *TCGA-LUAD* dataset, somatic mutation data were retrieved from the UCSC Xena multi-omics database platform (https://xenabrowser.net/datapages/?dataset=TCGA.LUAD.sampleMap%2Fmutation_broad&host=https%3A%2F%2Ftcga.xenahubs.net&removeHub=https%3A%2F%2Fxena.treehouse.gi.ucsc.edu%3A443) and preprocessed at the Broad Institute Genome Sequencing Center (22). WES data were generated on the Illumina platform. Mutation calls were calculated using the MuTect method (23), and only calls with variant allele frequency (VAF) >4.0% were included (22). The R package “maftools” (24) was then used to calculate the total number of somatic non-synonymous point mutations within each sample.

### RNA-seq and Gene Set Enrichment Analysis (GSEA)

For three immunotherapeutic cohorts, RNA-seq data were not available. For *TCGA-LUAD* datasets, RNA-seq data were assessed using the Illumina RNA sequencing platform. We downloaded the level 4 gene expression data from the UCSC Xena platform (22). The pre-processing and quality control of expression data have previously been described (22). The unit of mRNA expression value is pan-cancer normalized log_2_ (norm_count+1).

For pathway enrichment analysis, we used MSigDB (Molecular Signatures Database) of KEGG gene sets (25) to enrich the significant pathways, which were determined by a list of genes that highly correlated with increased TMB (Table S2; AUC > 0.65, *P* < 0.0001). For the enriched results, a *P* > 0.05 was considered statistically significant. We also recruited another tool, GSEA software (http://software.broadinstitute.org/gsea/index.jsp) (25), to confirm the pathway we enriched. GSEA integrates the expression data with phenotypes' information to determine whether a gene set significantly correlates with a defined phenotype. The normalized enrichment score (NES) and the nominal *P*-value are two primary statistics to examine the GSEA results. The ranking metric score is used to measure the correlation of a gene with a phenotype, with a positive value indicating a correlation with the first phenotype and a negative value indicating a correlation with the second.

### Mutational Signature Analysis

We used the “SignatureAnalyzer” R package (26, 27) to calculate the percentage of mutation signature within each tumor sample. “SignatureAnalyzer” can capture the non-negative matrix factorization algorithm (NMF) to decipher mutation signatures within cancer genomes, and then it automatically calculates the optimal number of mutation signatures (W) and the fraction of mutation signature in an individual sample. Mutation Annotation Format (MAF) files are available in the *TCGA-LUAD* dataset (http://gdac.broadinstitute.org/) and necessary for this analysis. The detailed method of mutation signature analysis has been described (https://software.broadinstitute.org/cancer/cga/msp).

### Immune Cellular Infiltration Estimates

The abundance of tumor-infiltrating immune cells (CD8+ T cells, T-regulatory cells, and macrophages) in LUAD samples was assessed using the CIBERSORT algorithm (28). CIBERSORT is an influential deconvolution method that uses support vector regression to quantify the cellular components from bulk tissue gene expression profiles. Based on gene expression data, CIBERSORT can accurately estimate the immune composition within a given tumor sample. We extracted the relative proportion of immune cells of *TCGA-LUAD* samples from the Pan-Cancer Atlas (https://www.cell.com/pb-assets/consortium/PanCancerAtlas/PanCani3/index.html) (18) and then compared them according to the indicated *MSH2* expression status.

### Statistical Analyses

Statistical analyses were performed using R software (version 3.5.2) and GraphPad Prism software (version 7.0.0). Student's *t*-test or Mann-Whitney *U* test was used to determine the differences between two groups. Kruskal-Wallis test was used to determine the differences among three or more groups. We used ROC curves with the highest Youden index to determine the optimum cut-off of TMB and *MSH2* expression.

The proportion of gene mutation was compared using Fisher's exact test. Pairwise correlations were calculated using the Spearman correlation formula. Multivariate logistic and linear regression models were conducted to assess the impact of gene expression on TMB, adjusting for other covariates described. All reported *p*-values were two-sided.

## Results

### Clinical and Genomic Characteristics of Selected Cohorts

**Figure 1:**
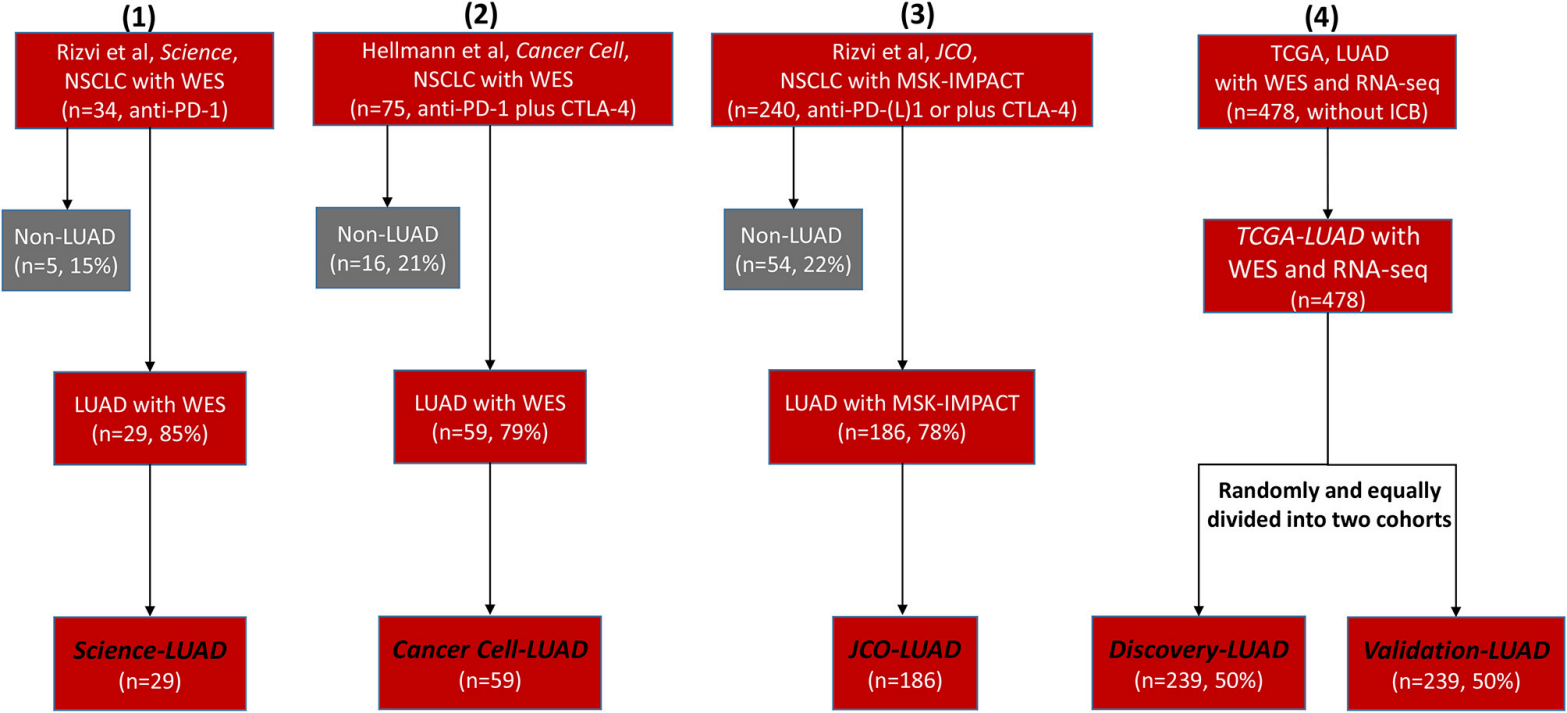
Flowchart of four selected clinical cohorts for statistical analysis. NSCLC, non–small cell lung cancer; WES, Whole-Exome Sequencing; LUAD, lung adenocarcinoma; PD-1, programmed cell death-1; CTLA-4, cytotoxic T lymphocyte antigen-4; MSK-IMPACT, Memorial Sloan Kettering-Integrated Mutation Profiling of Actionable Cancer Targets; TCGA, The Cancer Genome Atlas; ICB, immune checkpoint blockade.

We retrieved many previous studies and cancer databases, only collecting four high-quality LUAD datasets that contained both clinical and genomic information: 29 LUAD patients treated with anti-PD-1 therapy (*Science-LUAD*) (1), 59 LUAD patients treated with PD-1 plus CTLA-4 blockade (*Cancer Cell-LUAD*) (2), 186 LUAD patients treated with anti-PD-1/PD-L1 therapies or in combination with anti-CTLA-4 therapy (*JCO-LUAD*) (3), and 478 LUAD patients without immunotherapy (*TCGA-LUAD*) (Figure 1; Table S1). Pre-therapy tissues from LUAD patients were assessed by whole-exome sequencing (WES) in *Science-LUAD, Cancer Cell-LUAD*, and *TCGA-LUAD* cohorts while targeted panel sequencing (MSK-IMPACT panel, covering specific cancer-related genes) in *JCO-LUAD* dataset (Figure 1; Table S1). Since *TCGA-LUAD* datasets had more samples than the other three cohorts, we randomly divided it into two independent cohorts to further validate our hypothesis (*Discovery-LUAD* and *Validation-LUAD*, respectively) (Figure 1; Table S1).

**Figure 2:**
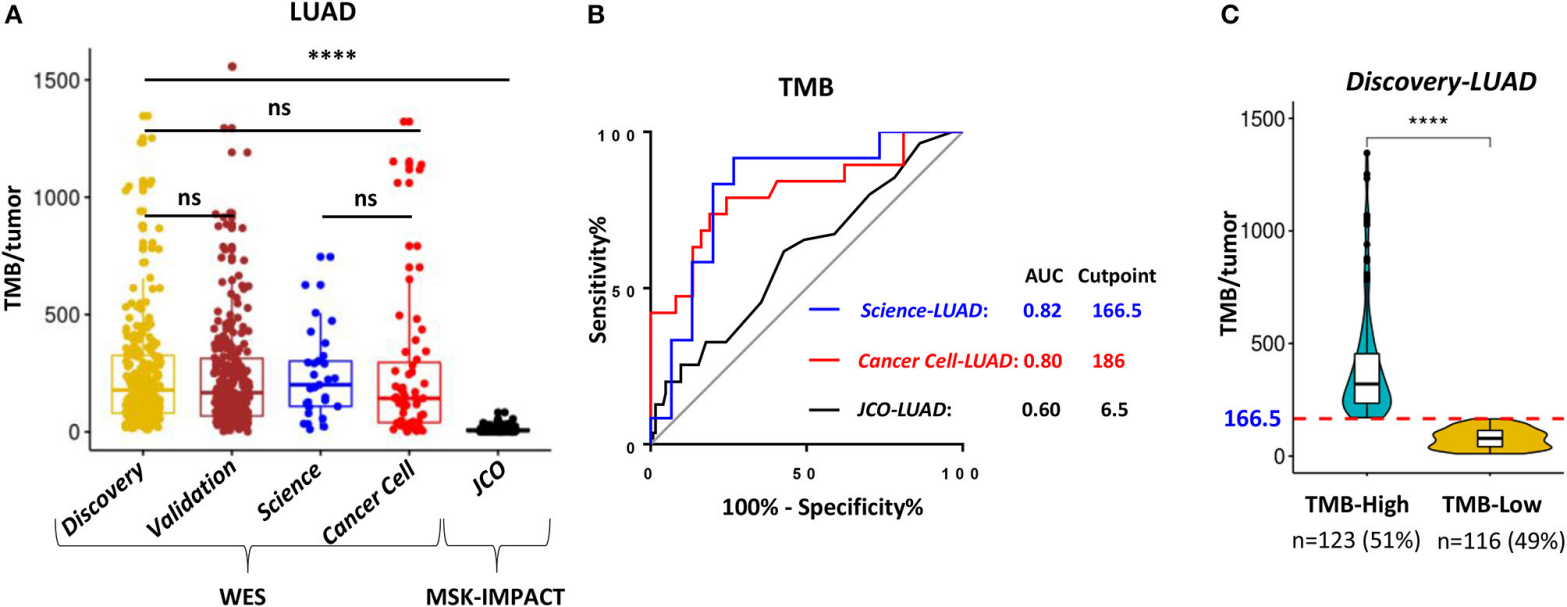
WES outperformed targeting-sequencing for assessing TMB as an ICB biomarker. **(A)** Quantitative analysis of TMB between *TCGA- LUAD* and the other three published LUAD cohorts for immunotherapy. **(B)** Receiver operating characteristic (ROC) curves for the correlation of TMB with clinical response to ICB therapies in the three cohorts. AUC, area under a ROC curve; Cutpoint, the Youden index-associated cutoff value of TMB. **(C)** Samples in the *Discovery-LUAD* cohort were stratified into two groups based on the Youden index-associated cutpoint of TMB from the *Science-LUAD* cohort. TMB-High: ≥166.5; TMB-Low: <166.5. ****P < 0.0001, ns, non significant.

TMB was defined as the total number of somatic non-synonymous point mutations. Except for the *JCO-LUAD* cohort [median six and interquartile range (IQR) 3-11] assessed by targeted panel sequencing, the quantity and range of TMB in *TCGA-LUAD* (median 178 and IQR 80-326 in the *Discovery-LUAD* cohort; median 167 and IQR 68-313 in the *Validation-LUAD* cohort) were similar to that in *Science-LUAD* (median 201 and IQR 109-302) and *Cancer Cell-LUAD* (median 143 and IQR 40-296) cohorts (Figure 2A), suggesting the homogeneity of these cohorts as previously reported (1, 2).

### WES Outperformed Targeted Panel Sequencing for Assessing TMB as an ICB Biomarker

Previous studies reported that TMB assessed by WES or targeted panel sequencing was significantly associated with improved efficacy of ICB therapies in LUAD (1–3). Moreover, the assessment of TMB by targeted panel sequencing also highly correlated with WES (*r* = 0.86, *P* < 0.001) (3). However, using receiver operator characteristic (ROC) curves as previously suggested (1–3, 16), we found that WES-based TMB achieved consistently better performance than targeted panel sequencing for predicting ICB response in LUAD (Figure 2B; AUC = 0.82 (*Science-LUAD*), 0.80 (*Cancer Cell-LUAD*), and 0.60 (*JCO-LUAD*), respectively). Additionally, TMB assessed by WES [Figure 2B; AUC = 0.82 (*Science-LUAD*); and 0.80 (*Cancer Cell-LUAD*), respectively] also performed better than PD-L1 expression detected by IHC for predicting ICB response in LUAD [Figure S1A; AUC = 0.61 (*Cancer Cell-LUAD*); and 0.69 (*JCO-LUAD*), respectively].

### *MSH2* Expression Significantly Correlated With Increased TMB and Performed Better Than *PD-L1* on Predicting TMB in LUAD

Given that transcriptomic data in three immunotherapeutic cohorts were not available, we turned to use multidimensional *TCGA-LUAD* datasets, which contained both genomic and transcriptomic features, to further explore the potential determinants associated with increased TMB in LUAD.

To demonstrate the potential clinical usefulness of TMB for predicting ICB response in LUAD, the Youden index was used to choose the optimum cut point of TMB (16, 29). The index-associated cut point of TMB in *Science-LUAD* was very close to that in the *Cancer Cell-LUAD* cohort (Figure 2B; TMB = 166.5 and 186, respectively), which was also very approximate to a previous report in NSCLC (TMB = 178) (1). Given that *Science-LUAD* cohort was only treated with PD-1 blockade and performed better than *Cancer Cell-LUAD* cohort on predicting ICB efficacy (Figure 2B; AUC = 0.82 and 0.80, respectively), we stratified the *Discovery-LUAD* cohort into two groups based on the TMB cutoff from *Science-LUAD* cohort (Figure 2C; TMB = 166.5).

**Figure 3:**
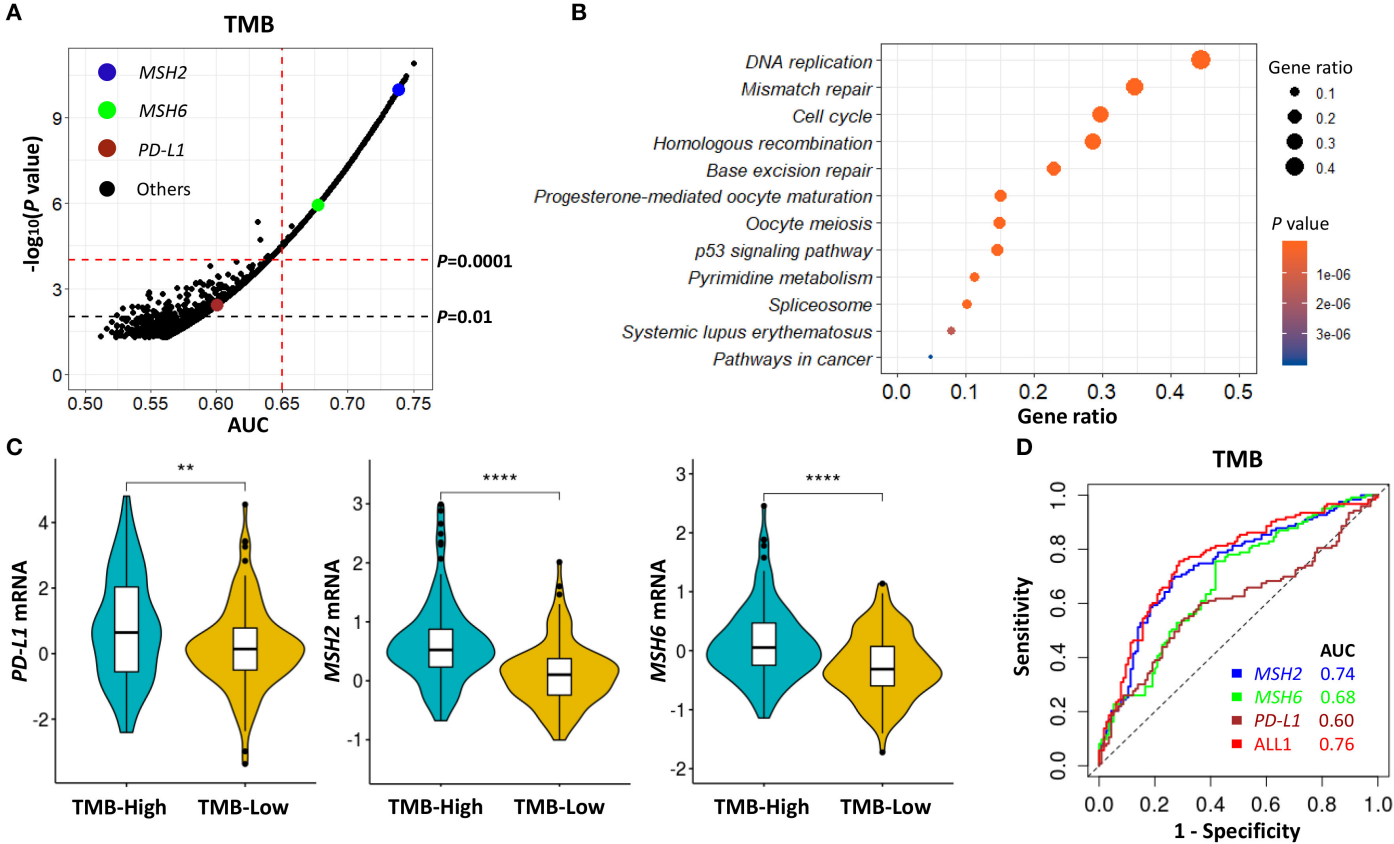
*MSH2* expression highly correlated with increased TMB and performed better than *PD-L1* on predicting TMB in the *Discovery-LUAD* cohort. **(A)** Volcano plot of AUC and rank sum *P-*values demonstrating the positive correlation of transcriptomic features with increased TMB in the *Discovery-LUAD* cohort. AUC, area under a ROC curve. **(B)** Pathway enrichment analysis of genes whose expression significantly and positively associated with increased TMB (AUC > 0.65, *P* < 0.0001), with *P* > 0.05 considered statistically significant. **(C)** Quantitative analysis of *PD-L1, MSH2*, and *MSH6* mRNA expression in two groups according to indicated TMB status. **(D)** ROC curves for the association of *PD-L1, MSH2*, and *MSH6* mRNA expression with TMB status. ALL1: the combination of *PD-L1, MSH2*, and *MSH6* mRNA expression. ****P < 0.0001, **P < 0.01.

According to the above TMB-defined groups in the *Discovery-LUAD* cohort, we performed the ROC test to all genes, examining the association of TMB with all transcriptomic features (Figure 3A). A list of genes, which highly and positively correlated with increased TMB (AUC > 0.65, *P* < 0.0001), were significantly enriched in the mismatch repair (MMR) pathway (Figures 3A,B; Tables S2, S3), consistent with the result of gene set enrichment analysis (GSEA) (Figure S2A). Notably, *MSH2* and *MSH6* are two key cancer-related MMR genes and were as similar as *PD-L1* expression significantly up-regulated in patients with high TMB in *Discovery-LUAD* cohort (Figures 3A–C; Table S3). These results could be reasonably speculated that patients with high TMB would potentially accelerate the expression of MMR-related genes to repair the impaired genome.

Additionally, we also examined the impact of *MSH2* and *MSH6* expression on TMB in the context of *PD-L1* expression. There were moderate correlations of TMB with *MSH2* and *MSH6* expression (Figure S3A; *r* = 0.46 and 0.39, respectively) while no significant association with *PD-L1* expression (Figure S3A; *r* = 0.13). In multivariate analysis incorporating *MSH2, MSH6*, and *PD-L1* expression, *MSH2* expression was the most robust gene associated with increased TMB in the *Discovery-LUAD* cohort (Figure 3D; Figures S3B,C). Of note, the ROC test incorporating *MSH2, MSH6*, and *PD-L1* expression did not significantly improve the predictive ability for TMB compared with *MSH2* expression alone [Figure 3D; AUC = 0.74 (*MSH2*) and 0.76 (ALL1), respectively].

### *MSH2* Expression Outperformed Other MMR-Related Genes for Predicting TMB in LUAD

The MMR pathway is crucial for maintaining genomic integrity, and the deficiency of MMR (MMRd) is also highly sensitive to ICB therapies (11, 30). The potential mechanism is that tumors with MMRd can result in microsatellite instability (MSI) and are a specific subset of high TMB tumors (5). However, in LUAD, the positivity rate of MMRd/MSI assessed by genomic variations is <1% and much lower than the objective response rate to PD-1 blockade in unselected patients (13, 19, 30–33). Given that previous studies were mainly focusing on genomic variations to explore the MMRd mechanism in LUAD (13, 33) and our data showed that *MSH2* expression was strongly associated with increased TMB in *Discovery-LUAD* cohort (Figure 3; Figure S3), we further examined the transcriptome-based MMRd status in LUAD patients with high TMB.

**Figure 4:**
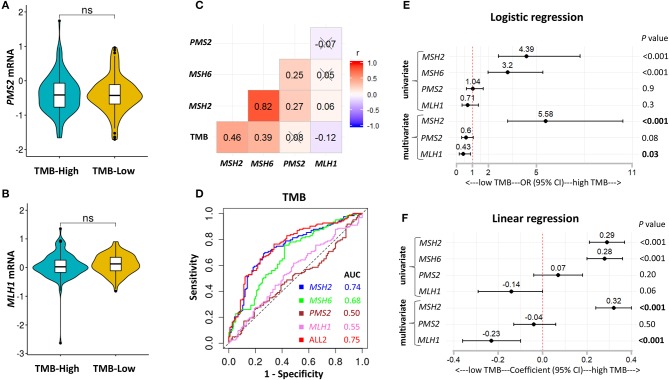
*MSH2* expression outperformed other MMR-related genes for predicting TMB in the *Discovery-LUAD* cohort. **(A,B)** Quantitative analysis of *PMS2* and *MLH1* mRNA expression in two groups based on TMB status. **(C)** Pairwise correlations between TMB and four MMR-related gene expression. Cross indicated no significant correlation (*P* > 0.05); r: Spearman correlation coefficient. **(D)** ROC curves for the association of *MSH2, MSH6, PMS2*, and *MLH1* mRNA expression with TMB status. ALL2: the combination of *MSH2, MSH6, PMS2*, and *MLH1* mRNA expression. **(E,F)** Forest plots for univariate and multivariate logistic or linear analysis of TMB with indicated gene expression. Of note, *MSH6* expression was not analyzed in multivariate regression model given its tight correlation with *MSH2* expression. OR, odds ratio. ns, not significant.

*MSH2, MSH6, PMS2*, and *MLH1* are four genes that play a critical role in DNA MMR (13, 30). Four proteins codified by these genes function in heterodimer pairs (MSH2-MSH6 and MLH1-PMS2) to preserve genomic integrity (13, 30). In clinical practice, the inactivation of one of the four genes detected by next-generation sequencing (NGS) or IHC suggests an MMRd mechanism within a tumor (13, 30). However, MSH2 and MLH1 are obligatory partners for forming the two heterodimers, while MSH6 and PMS2 can be replaced by other MMR proteins, such as MSH3, PMS1, and MLH3 (13, 30). We observed that *MSH2* was significantly mutated in patients with high TMB, but it only accounted for 5.7% of high TMB tumors in LUAD (Figures S4A–D). In addition, except for the other two MMR genes, *MLH1* and *PMS2* were not significantly up-regulated in patients with high TMB (Figures 3C, 4A,B), suggesting a low abundance of MLH1-PMS2 heterodimers existed in high TMB tumors. Furthermore, *MSH2* whose expression displayed the strongest correlation with increased TMB than the other three MMR genes (Figure 4C), and the ROC test incorporating *MSH2, MSH6, PMS2*, and *MLH1* expression did not significantly improve the predictive ability for TMB compared with *MSH2* expression alone [Figure 4D; AUC = 0.74 (*MSH2*); and 0.75 (ALL2), respectively].

Interestingly, using both multivariate logistic and linear regression analysis, we revealed that TMB displayed a significantly positive association with *MSH2* expression while an inverse correlation with *MLH1* expression in *Discovery-LUAD* cohort (Figures 4E,F). Furthermore, GSEA incorporating all MMR-related genes also confirmed these findings (Figures S2B,C). These results suggested that down-regulated *MLH1* expression in patients with high TMB might result in the dysfunction of the MMR machinery and then potentially facilitate the accumulation of mutations in LUAD.

**Figure 5:**
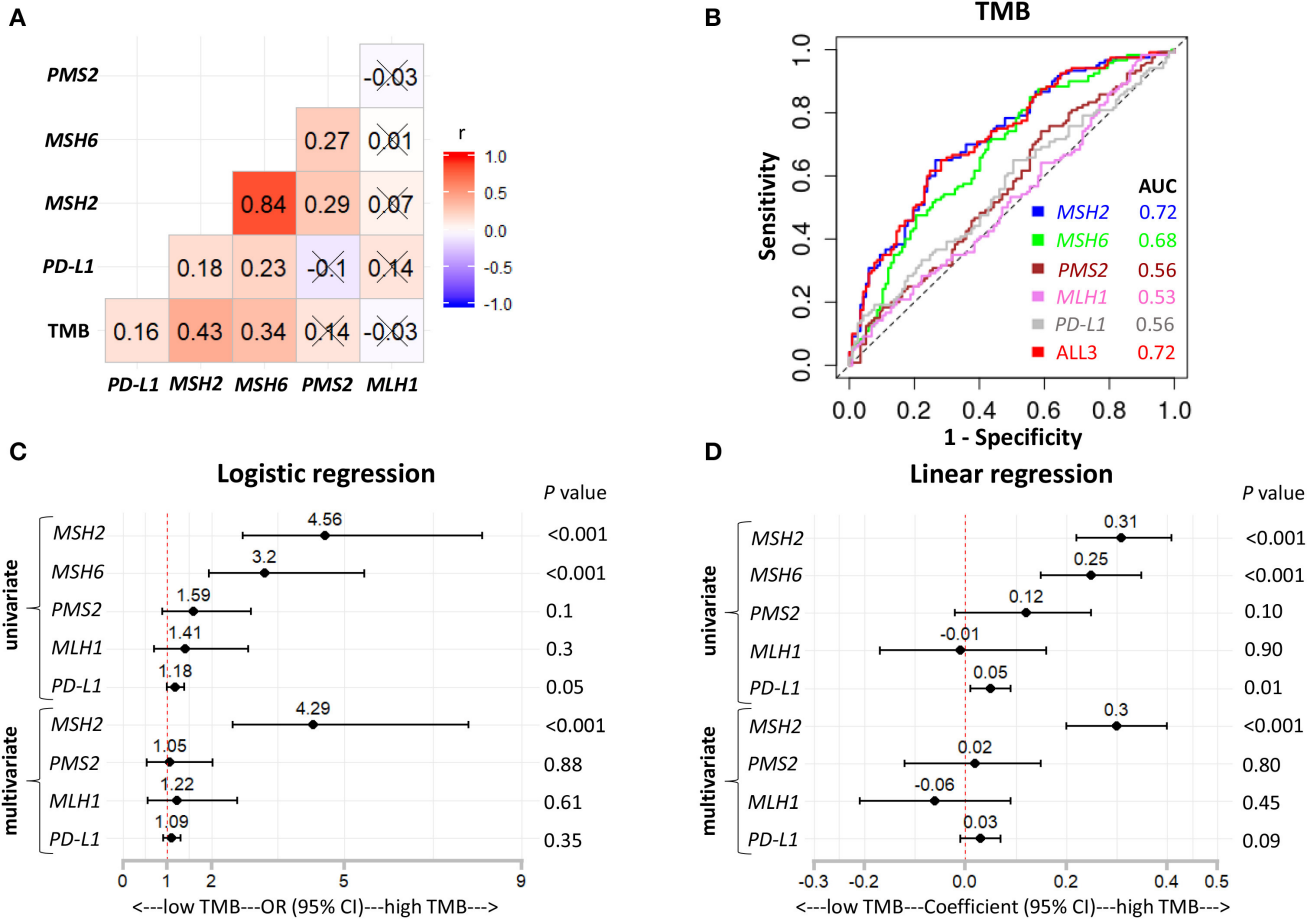
*MSH2* expression outperformed other indicated genes for predicting TMB in the *Validation-LUAD* cohort. **(A)** Pairwise correlations between TMB and the indicated gene expression. Cross indicated no significant correlation (*P* > 0.05); r: Spearman correlation coefficient. **(B)** ROC curves for the association of *PD-L1, MSH2, MSH6, PMS2*, and *MLH1* mRNA expression with the TMB status. ALL3: the combination of *PD-L1, MSH2, MSH6, PMS2*, and *MLH1* mRNA expression. **(C,D)** Forest plots for univariate and multivariate logistic or linear analysis of TMB with the indicated gene expression. Of note, *MSH6* expression was not analyzed in multivariate regression model given its high correlation with *MSH2* expression. OR, odds ratio.

To further consolidate and extend our findings, we performed two additional analyses. First, we used multivariate regression analysis to demonstrate that *MSH2* expression was independently associated with increased TMB, with adjustment for patients' sex, age, and pack-year (smoking index) in the *Discovery-LUAD* cohort (Figures S5A,B). Second, we validated the hypothesis that *MSH2* expression was the strongest determinant associated with increased TMB in another independent *Validation-LUAD* cohort (Figures 5A–D).

### *MSH2* Expression Outperformed Other MMR-Related Genes for Predicting Smoking Signature in LUAD

**Figure 6:**
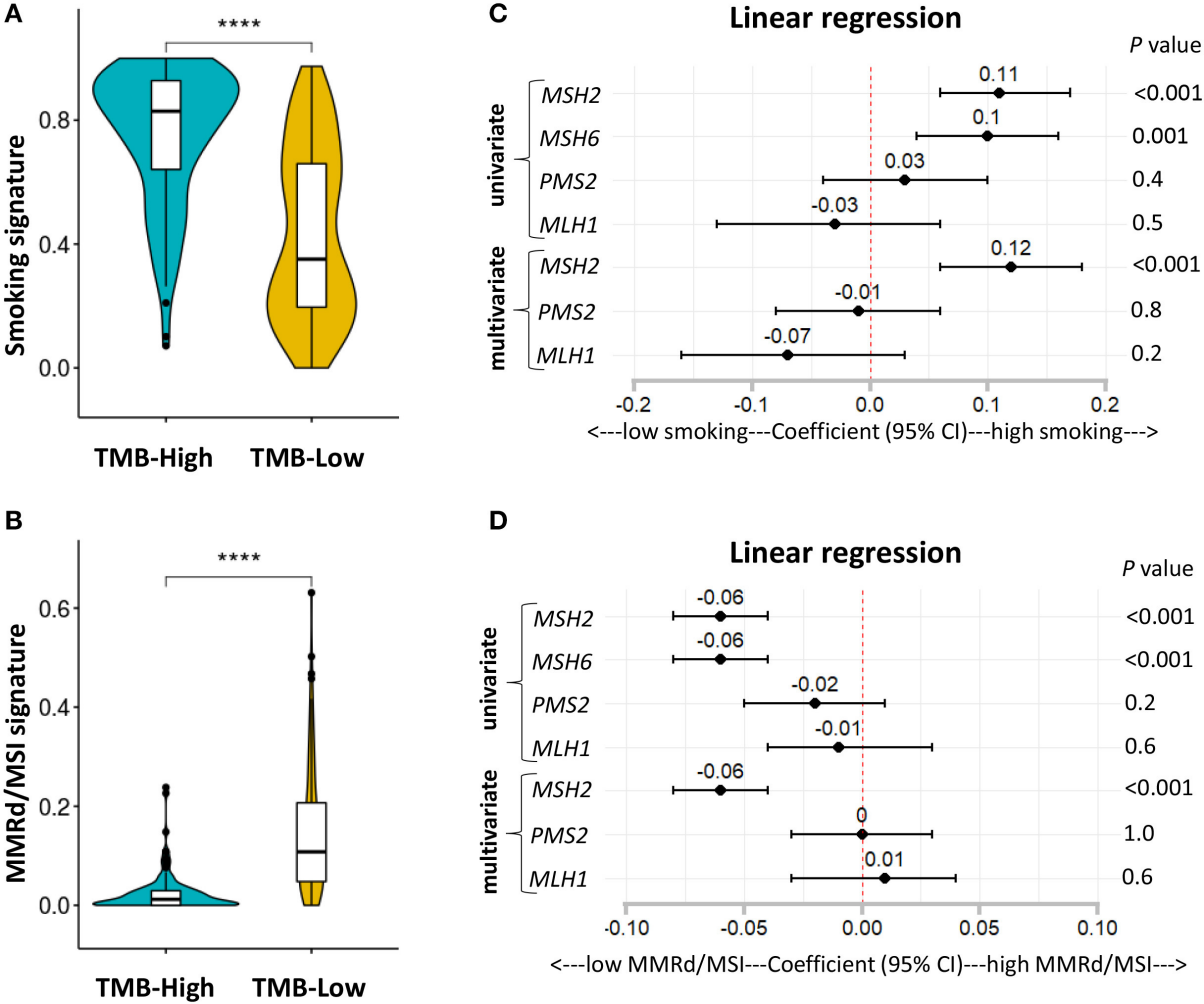
*MSH2* expression positively correlated with smoking signature but negatively associated with MMRd signature in the *Discovery-LUAD* cohort. **(A,B)** Quantitative analysis of smoking and MMRd/MSI signature in two groups based on the TMB status. **(C)** Forest plot for univariate and multivariate linear analysis of smoking signature with the four MMR-related gene expression. **(D)** Forest plot for univariate and multivariate linear analysis of MMRd/MSI signature with the four MMR-related gene expression. Of note, *MSH6* expression was not analyzed in multivariate regression model given its tight correlation with *MSH2* expression. ****P < 0.0001.

It is well-known that LUAD exhibiting high TMB is strongly associated with cigarette smoking, and smoking signature is also highly sensitive to ICB therapies in LUAD (1, 26). Consistent with previous studies (1, 26), we found that patients with high TMB significantly increased the fractions of smoking signature in LUAD (Figure 6A; Figures S6A–D). However, MMRd/MSI signature, as determined by NGS data, displayed significantly decreased proportions in patients with high TMB (Figure 6B; Figures S6A–D), suggesting that genome-assessed MMRd/MSI signature was not suitable as a potential predictor of increased TMB and improved ICB efficacy in LUAD.

Furthermore, using multivariate regression analysis, we demonstrated that *MSH2* expression was the most robust MMR feature positively associated with smoking signature while inversely correlated with MMRd/MSI signature in *Discovery-LUAD* cohort (Figures 6C,D), suggesting that high *MSH2* expression might also be a potential predictor of increased smoking signature in LUAD.

### High Expression of *MSH2* Significantly Correlated With Increased *PD-L1* Expression and CD8+ T Cell Infiltration Within the Tumor Microenvironment

**Figure 7:**
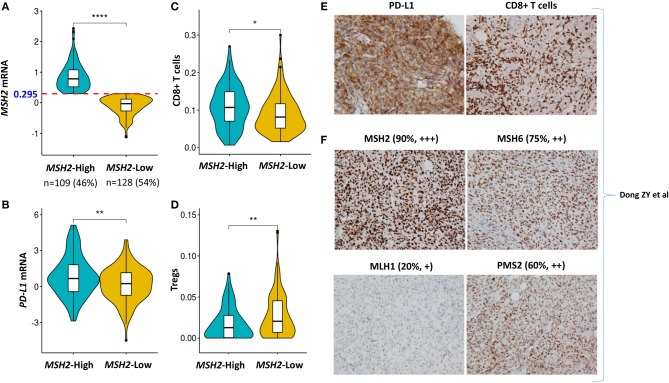
High expression of *MSH2* highly correlated with increased *PD-L1* expression and CD8+ T cell infiltration in the *Validation-LUAD* cohort. **(A)** Samples in the *Validation-LUAD* cohort were stratified into two groups based on the Youden index-associated cutoff of *MSH2* expression from Figure 5B. *MSH2*-High: ≥0.295 (*n* = 109); *MSH2*-Low: <0.295 (*n* = 128); two samples whose expression profiles were missing. **(B–D)** Quantitative analysis of *PD-L1* expression and the infiltration of immune cells in individual tumor tissues based on *MSH2* expression. **(E,F)** IHC staining of PD-L1, CD8, and four MMR- related genes in one LUAD patient who derived durable clinical benefit from anti-PD-1 therapy. raw IHC data retrieved from Dong et al. (10). ****P < 0.0001, **P < 0.01, *P < 0.05.

PD-L1 expression and the infiltration of CD8+ T cells are two important biomarkers for assessing the immunotherapeutic microenvironment in LUAD (1, 5, 10, 12). Therefore, we further examined the association of *MSH2* expression with *PD-L1* expression and CD8+ T cell infiltration within the tumor microenvironment. We stratified the *Validation-LUAD* samples into two groups according to the Youden index-associated cutoff value of *MSH2* expression (Figure 7A). We revealed that patients with high *MSH2* expression significantly increased *PD-L1* expression and CD8+ T cell infiltration while decreased the infiltration of T-regulatory cells (Tregs) (Figures 7B–D). It has been reported that tumor-associated macrophages (TAMs) were also important for assessing the efficacy of anti-PD-1/PD-L1 therapies (34). We found that inflammatory M1 macrophages, but not pro-tumor M2, were also significantly infiltrated into the tumor tissues with high *MSH2* expression (Figures S7A,B). These results suggested that patients with high *MSH2* expression displayed a significant immunotherapy-responsive microenvironment in LUAD.

Of particular note, one LUAD patient who derived durable clinical benefit from anti-PD-1 therapy showed the strong staining of both PD-L1 expression and CD8+ T cell infiltration. Moreover, this patient also displayed the strongest staining of MSH2 expression among the four key MMR proteins, which directly supported our hypothesis that *MSH2* expression might be a potential surrogate biomarker of TMB to predict ICB response in LUAD [Figures 7E,F; raw IHC data retrieved from Dong et al. (10)].

## Discussion

ICB-based therapies targeting CTLA-4 or PD-1 have shown a promising future in multiple cancer types, but the molecular mechanism between them is completely different (35). Additionally, anti-PD-1 therapy performs much better than anti-CTLA-4 therapy on the efficacy, survival, and adverse events (5, 35). Therefore, this study mainly focused on LUAD for anti-PD-1 therapy.

TMB is one of the most important biomarkers for predicting ICB response in NSCLC (1–3, 7), and it also shows predictive efficacy for ICB therapies in other types of solid tumors (7, 36), but it still has some limitations (5, 6, 14, 19). For example, the cut-offs of TMB for identifying ICB responders are different for different tumor types, and the test platform for assessing TMB has also not been standardized (5, 14, 19). Thus, more studies are turning to develop surrogate biomarkers that highly correlate with the TMB status, such as genetic mutations of DNA damage response pathways and *TP53/KRAS* (10, 19, 37). However, these mutations are positive for only a minority of patients, and the broad detection of these TMB-related gene mutations in clinical practice remains challenging (19). Titin (*TTN*) is the longest gene within the whole genome, and its mutations have also been proposed as a surrogate TMB biomarker for predicting ICB response in solid tumors (19). However, *TTN* mutations are not the cause of high TMB in tumors, and its mutations also account for a small cohort of candidates (29.68%) (19, 38). In addition, targeted panel sequencing, such as MSK-IMPACT panel, has also been developed and approved by US Food and Drug Administration (FDA) to estimate TMB, but its predictive accuracy for ICB response is significantly lower than WES in LUAD (Figure 2B), which suggests that it still needs more optimizations. Moreover, blood-based TMB (bTMB) is being developed to predict ICB response in NSCLC, while blood samples for detecting bTMB must contain the mutated circulating tumor DNA (ctDNA) that must be shed from the tumor, which has limited its clinical application (39).

Unlike previous studies that focused on genomic variations (10, 19, 37, 40), we turned to the transcriptomic landscape to explore the potential surrogates of TMB in LUAD. We revealed that *MSH2* was the most robust MMR gene whose expression significantly correlated with increased TMB in LUAD. Mechanistically, given the low mutation rates of MMR-related genes in LUAD, the transcriptome-based dysfunction of the MMR machinery is more likely to be the cause of high TMB. Therefore, this study mainly focuses on MMR-related gene expression. The other nine genes (*CDCA5, MCM10, GINS4, KIAA1524, KIF2C, NUF2, CDC20, CDC7, THOC4*; Figure 3A; Table S2) showing better performance than *MSH2* may also be potential TMB indicators in LUAD, which still needs more mechanistic investigations.

MMRd testing has primarily been developed and tested in patients with colorectal and endometrial cancer to predict ICB response given their relatively high positive rates (13, 30, 33, 41). Mechanistically, tumors with MMRd are often hypermutated and can result in microsatellite instability (MSI) within the genome. Therefore, MSI has been proposed as a marker of MMRd in previous studies (13, 30, 33). However, in contrast to the findings in colorectal and endometrial cancer (13, 26), we found that MMRd/MSI signature was significantly low in LUAD with high TMB (Figure 6B; Figures S6A–D), suggesting that genome-based MMRd/MSI might not cause a high mutation load in LUAD. In addition, we observed that high *MSH2* expression showed a significantly inverse association with increased MMRd/MSI signature in LUAD (Figure 6D). Given that the relationships between MMRd/MSI and TMB are complex and different for different tumor types (13), more mechanistic investigations are required to illuminate these results in LUAD.

In previous studies (13, 30, 33), all four MMR proteins (MSH2, MSH6, PMS2, and MLH1) were always detected together by IHC to determine the MMRd status within a tumor. However, the IHC method was used to indirectly infer the mutation status of the four MMR genes (13, 30, 33). Of particular note, in LUAD, the IHC-based method has rarely been used to detect the MMRd status (13, 30, 33).

Importantly, our data revealed that a transcriptome-based, not genome-based, MMRd mechanism widely existed in LUAD, which might partly illuminate the cause of high TMB in LUAD. The potential mechanism is that both MSH2 and MLH1 proteins are essential partners for forming the MMR machinery (13, 30). However, we observed that TMB significantly and positively correlated with *MSH2* expression but inversely correlated with *MLH1* expression in LUAD (Figures 4E,F; Figures S2B,C). Moreover, *MLH1* was the only gene whose expression was significantly down-regulated in LUAD tissues compared with the other three MMR genes (Figures S8A–D). *MLH1* expression may be suppressed by its promoter methylation in LUAD (33, 42, 43). These results suggested that down-regulated *MLH1* expression might impair the MMR machinery to repair the damaged genome and then caused more mutation load in LUAD.

One limitation of this study is that we only collected one LUAD sample showing the direct evidence that MSH2 expression alone could be a surrogate TMB biomarker to predict ICB response in LUAD. Because of the lack of public LUAD data, we could not directly validate this result in a large cohort. However, more prospective clinical trials are required to validate this correlation. Another limitation is that the TMB cutoff (TMB = 166.5) for stratifying LUAD samples was based on a small number of samples, which still needs large cohorts to determine. However, given the intratumoral heterogeneity across different cancer subtypes (1–3, 7, 16, 17), our data are much more homogeneous and thus more reliable to find the specific biomarkers benefiting the specific patients. Additionally, we also recruited another independent cohort (*Validation-LUAD* cohort) to validate our conclusion and proposed a mechanistic connection between *MSH2* expression and increased TMB in LUAD.

Since ICB therapies are associated with specific adverse events, it is profound to identify predictive biomarkers to select patients who are more likely to derive the maximum benefits from ICB therapies. Therefore, more multi-omic datasets are indispensable to explore and improve the efficacy of immunotherapies. It is possible that *MSH2* expression can be applied jointly with other factors to acquire a greater prediction performance, which is already suggested that combining multiple biomarkers are more robust than a single analyte for predicting ICB efficacy (7, 10, 12, 17, 29, 39).

In summary, our data suggest that *MSH2* expression highly correlates with increased TMB and the immunotherapy-responsive microenvironment in LUAD. Prospective clinical trials are required to further confirm these results.

## Data Availability Statement

TCGA-LUAD datasets were extracted from the UCSC Xena multi-omics database platform (https://xenabrowser.net/datapages/).

## Author Contributions

MJ initiated and performed the analysis. MJ, LY, and QY wrote and revised the manuscript. TC supervised the project.

### Conflict of Interest

The authors declare that the research was conducted in the absence of any commercial or financial relationships that could be construed as a potential conflict of interest.
