# Supplementary material for: Association of MSH2 Expression With Tumor Mutational Burden and the Immune Microenvironment in Lung Adenocarcinoma
Source: Front Oncol. 2020 Feb 21;10:168. doi: 10.3389/fonc.2020.00168 (PMC7046689; doi:10.3389/fonc.2020.00168)
Supplement: Supplementary file 1 [file Data_Sheet_1.zip › Supplementary Materials 1.PDF]

Table S1

Table S1. Baseline clinical characteristics.

No. (%)

	Science-LUAD	*Cancer Cell-LUAD*	*JCO-LUAD*	*TCGA-LUAD*	*Discovery-LUAD*	*Validation-LUAD*
No. of patients	29	59	186	478	239	239
Age (years), median (range)	63(41-80)	63(42-87)	66(22-92)	67(38-88)	67(38-88)	67(40-87)
Gender						
Male	13(45)	22(37)	83(45)	221(46)	111(46)	110(46)
Female	16(55)	37(63)	103(55)	257(54)	128(54)	129(54)
Smoking status						
Ever	24(83)	46(78)	145(78)	169(35)	83(35)	86(36)
Never	5(17)	13(22)	41(22)	29(6)	14(6)	15(6)
Unknown	0(0)	0(0)	0(0)	280(59)	142(59)	138(58)
Histology						
LUAD	29(100)	59(100)	186(100)	478(100)	239(100)	239(100)
PD-L1 expression (IHC)				Not done	Not done	Not done
0%	4(14)	21(35)	36(19)			
>=1%	22(76)	34(58)	30(16)			
Unknown	3(10)	4(7)	120(65)			
Immunotherapy antibody	PD-1	PD-1 + CTLA-4	PD-(L)1	Not done	Not done	Not done
Mutation detection method	WES	WES	MSK-IMPACT	WES	WES	WES
Transcriptomic profiling	Not done	Not done	Not done	RNA-seq	RNA-seq	RNA-seq

WES, Whole-Exome Sequencing; LUAD, lung adenocarcinoma; PD-1, programmed cell death-1; CTLA-4, cytotoxic T lymphocyte antigen-4; MSK-IMPACT, Memorial Sloan Kettering-Integrated Mutation Profiling of Actionable Cancer Targets; TCGA, The Cancer Genome Atlas.

Figure S1

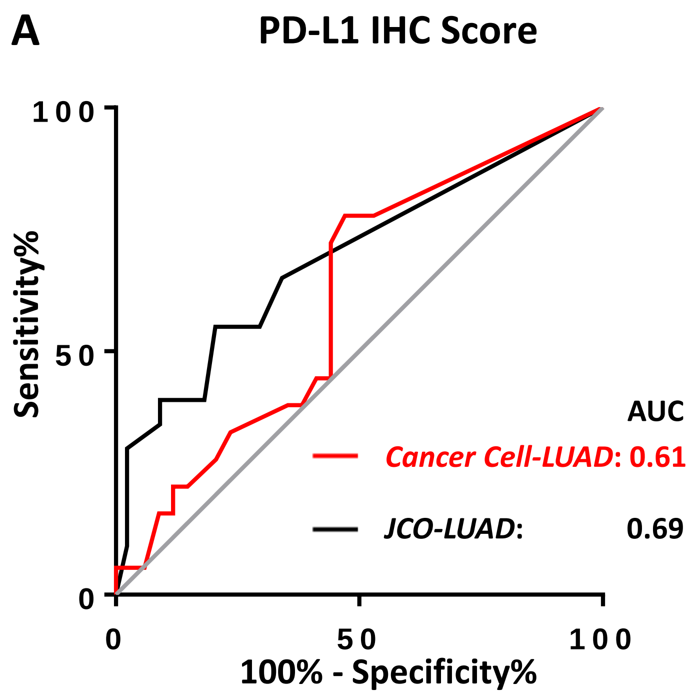

Figure S1. The performance of PD-L1 expression on predicting ICB response. (A) ROC curves for the correlation of PD-L1 expression with clinical response to ICB therapies in *Cancer Cell-LUAD* and *JCO-LUAD* cohorts. PD-L1 IHC score in *Science-LUAD* cohort was discrete and not available for ROC analysis.

Figure S2

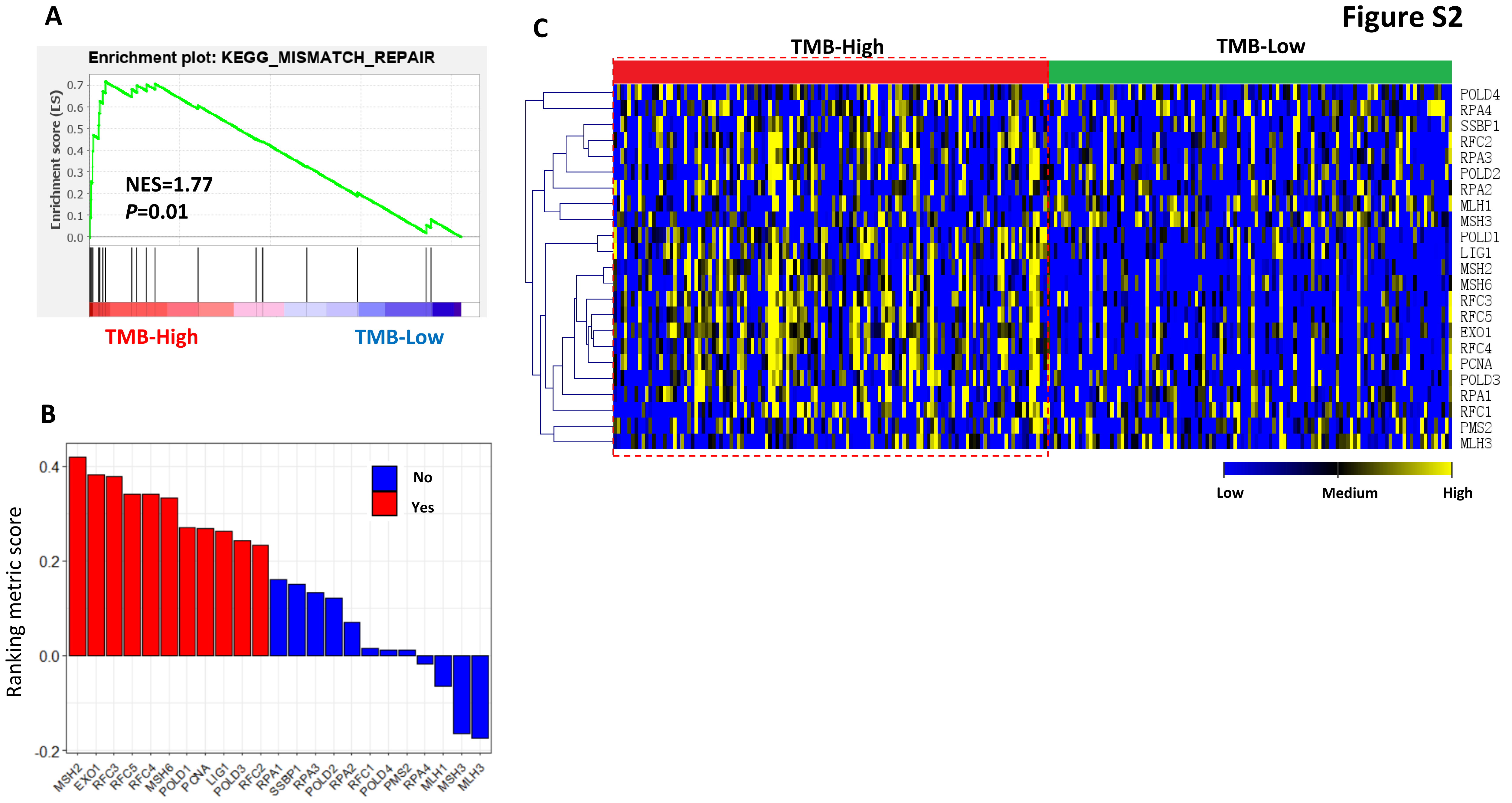
A
Enrichment plot: KEGG_MISMATCH_REPAIR
Enrichment score (ES)
0.7
0.6
0.5
0.4
0.3
0.2
0.1
0.0
NES=1.77
P=0.01
TMB-High
TMB-Low
B
Ranking metric score
0.4
0.2
0.0
-0.2
No
Yes
MSH2 EXO1 RFC3 RFC5 RFC4 MSH6 POLD1 PCNA LIG1 POLD3 RFC2 RPA1 SSBP1 RPA3 POLD2 RPA2 RFC1 POLD4 PMS2 RPA4 MLH1 MSH3 MLH3
C
TMB-High
TMB-Low
POLD4
RPA4
SSBP1
RFC2
RPA3
POLD2
RPA2
MLH1
MSH3
POLD1
LIG1
MSH2
MSH6
RFC3
RFC5
EXO1
RFC4
PCNA
POLD3
RPA1
RFC1
PMS2
MLH3
Low
Medium
High

Figure S2. MMR pathway was significantly enriched in high TMB group in the *Discovery-LUAD* cohort. (A) Gene set enrichment analysis (GSEA) revealed that MMR pathway was significantly enriched in patients with high TMB. NSE, normalized enrichment score. (B) The correlation of all MMR-related gene expression with the TMB status measured by the ranking metric score. A positive score indicates a correlation with the high TMB status and a negative score indicates a correlation with the low TMB status; The red indicates a gene that contributes most to the enrichment result and the blue indicates a gene that contributes less. (C) Heat map of all MMR-related gene expression between patients with high TMB and low TMB.

Figure S3

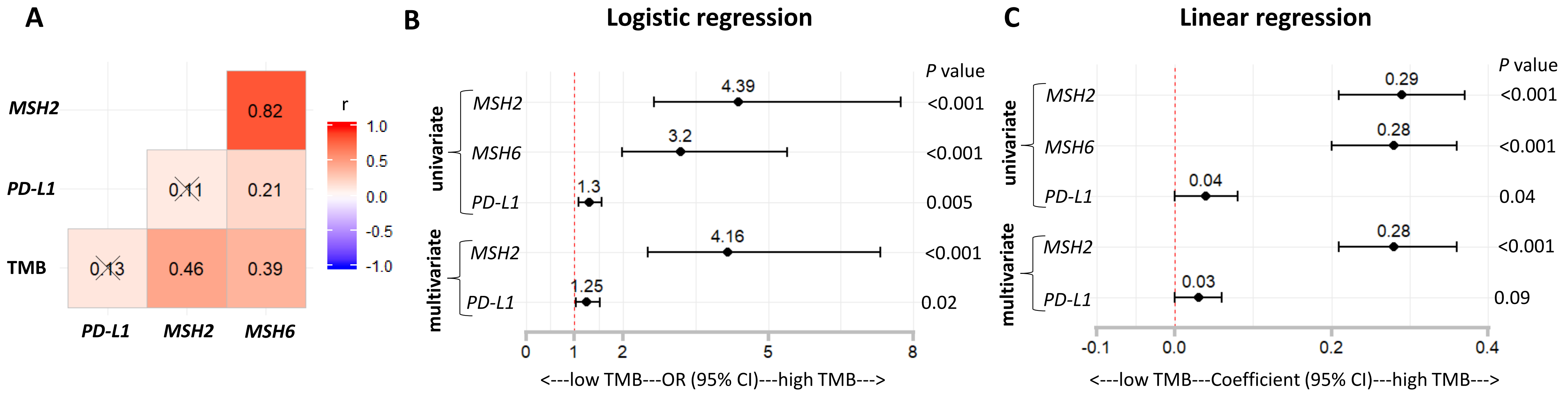

Figure S3. *MSH2* expression outperformed *PD-L1* expression for predicting TMB in the *Discovery-LUAD* cohort. (A) Pairwise correlations between TMB and the indicated gene expression. Cross indicated no significant correlation (*P*>0.05); r: Spearman correlation coefficient. (B and C) Forest plots for univariate and multivariate logistic or linear analysis of TMB with *PD-L1, MSH2*, and *MSH6* mRNA expression. Of note, *MSH6* expression was not analyzed in multivariate regression model given its tight correlation with *MSH2* expression. OR: odds ratio.

Figure S4

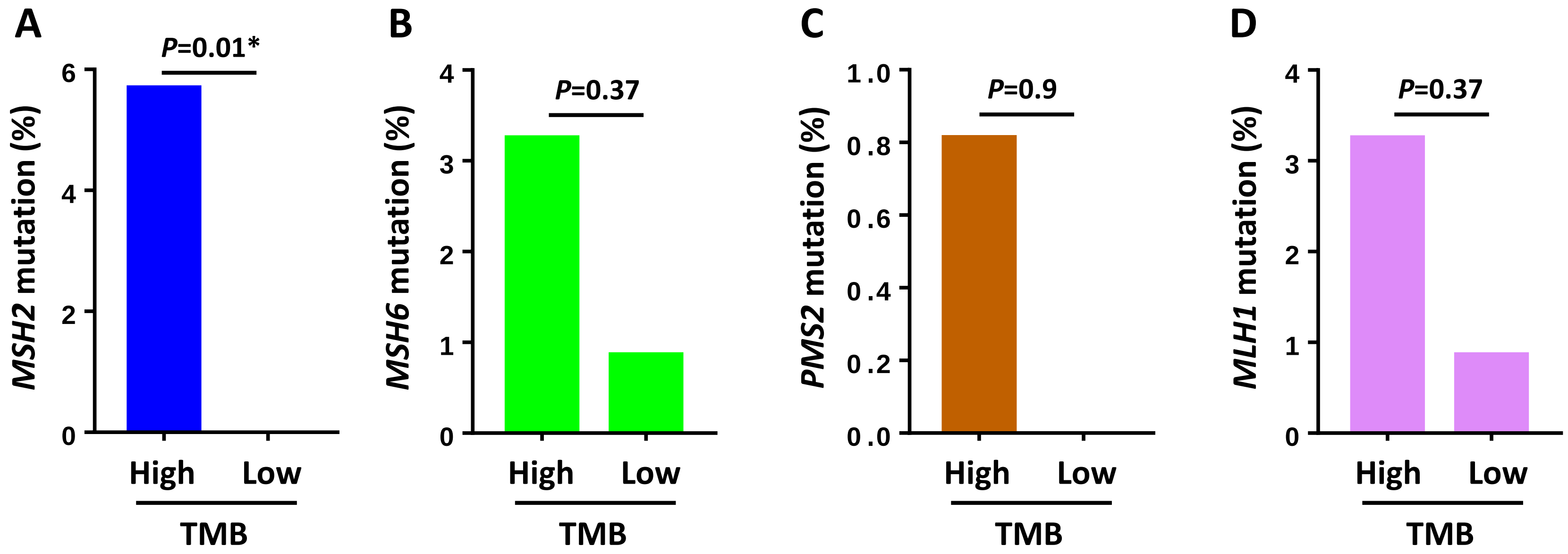

Figure S4. The mutation rates of four MMR-related genes according to indicated TMB status in the *Discovery-LUAD* cohort. (A-D) The proportion of *MSH2, MSH6, PMS2*, and *MLH1* nonsynonymous point mutations in two groups according to the indicated TMB status.

Figure S5

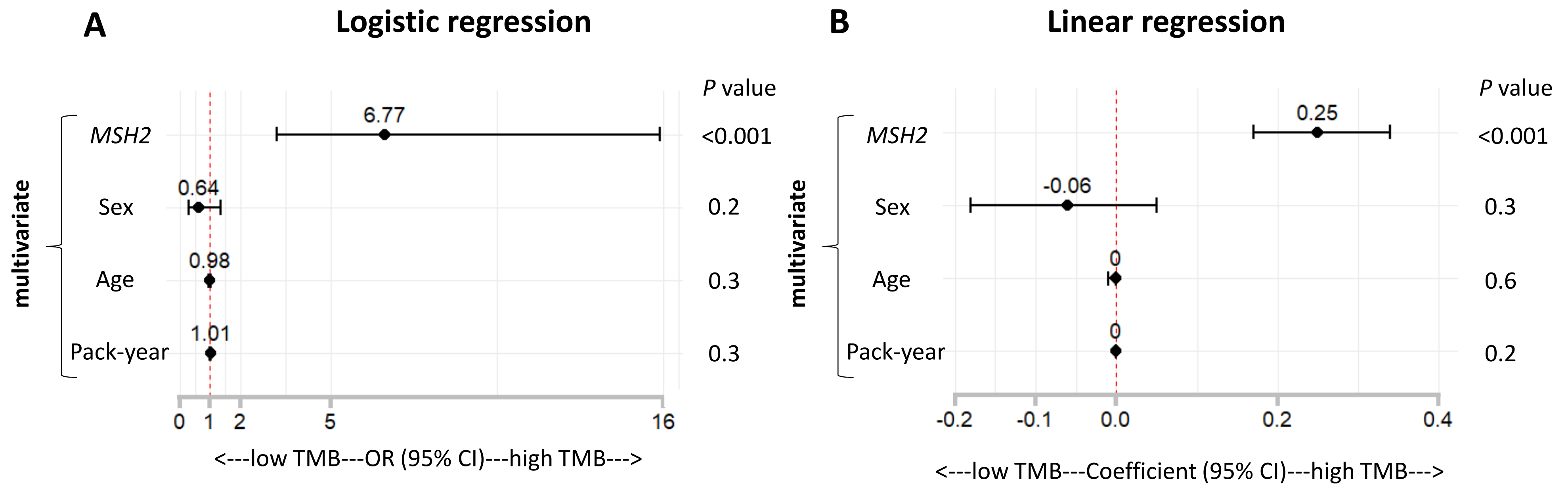

Figure S5. *MSH2* expression outperformed other features for predicting TMB in the *Discovery-LUAD* cohort. (A and B) Forest plots for multivariate logistic or linear analysis of TMB with *MSH2* mRNA expression, sex, age, and pack-year (smoking index). OR: odds ratio.

Figure S6

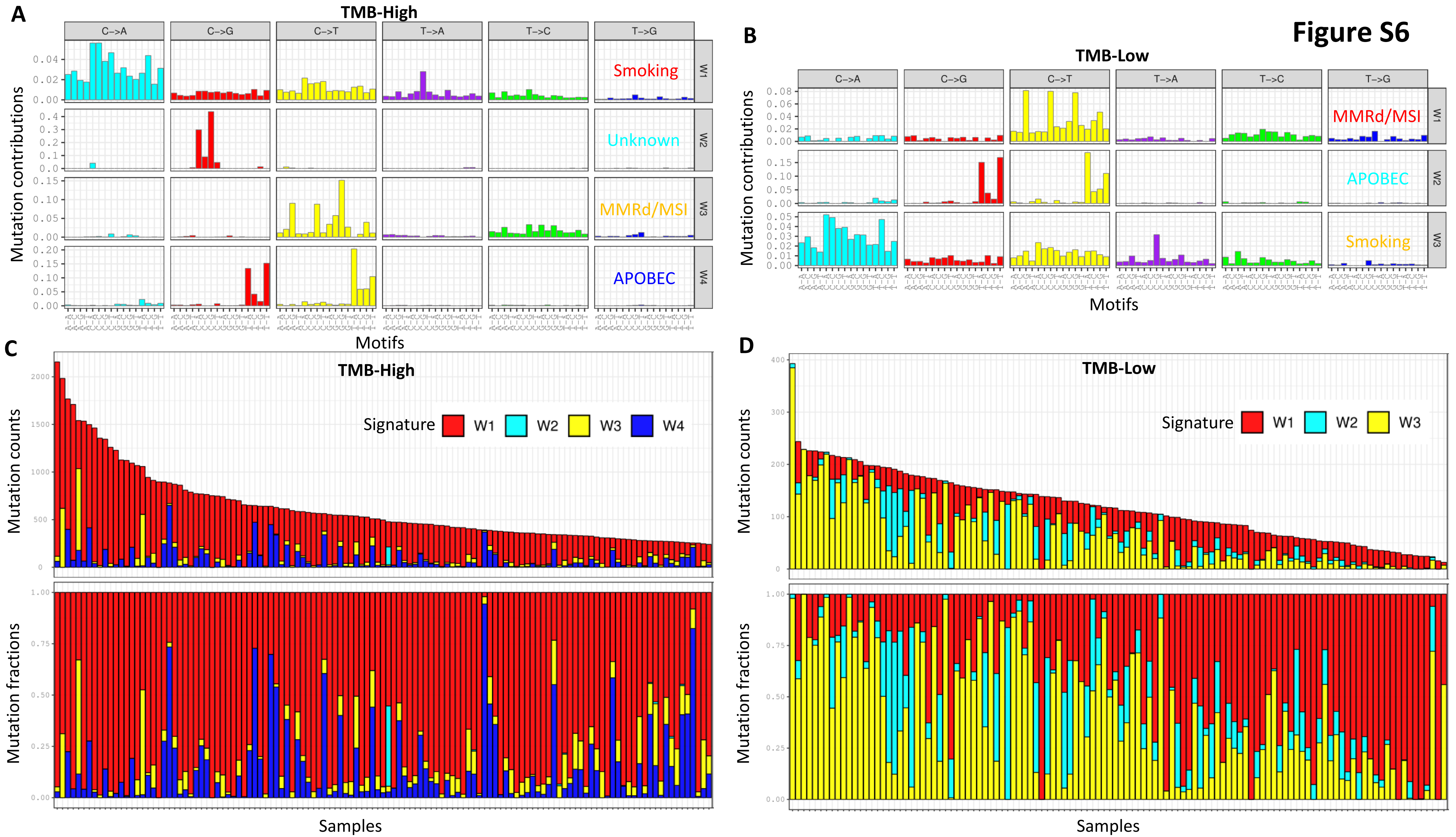
A
TMB-High
C->A
C->G
C->T
T->A
T->C
T->G
Smoking
Unknown
MMRd/MSI
APOBEC
W1
W2
W3
W4
Mutation contributions
Motifs
B
TMB-Low
MMRd/MSI
APOBEC
Smoking
Mutation contributions
Motifs
C
TMB-High
Signature
W1
W2
W3
W4
Mutation counts
Mutation fractions
Samples
D
TMB-Low
Signature
W1
W2
W3
Mutation counts
Mutation fractions
Samples

Figure S6. The landscape of mutation signature in the *Discovery-LUAD* cohort. (A and B) Mutation signatures were identified across 96 mutation contexts in two groups according to the indicated TMB status. (C and D) The contributions of each mutation signature to individual tumors.

Figure S7

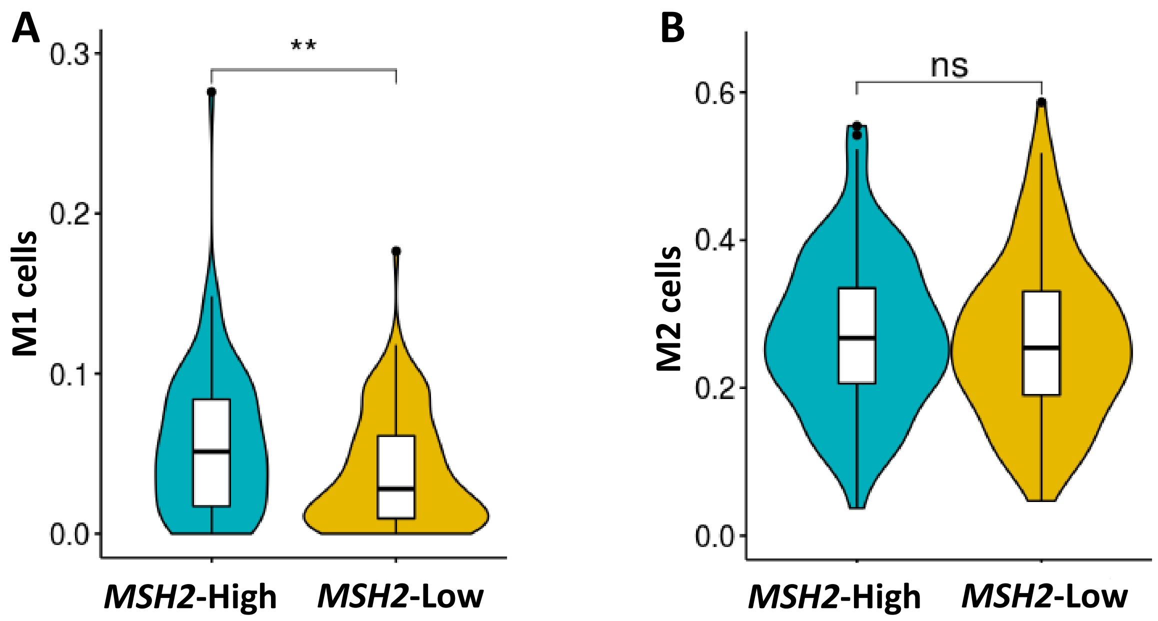

Figure S7. High expression of *MSH2* significantly correlated with increased macrophage M1 cell infiltration in the *Validation-LUAD* cohort. (A and B) Quantitative analysis of the infiltration of the subtype of macrophage cells in individual tumor tissues based on *MSH2* expression.

Figure S8

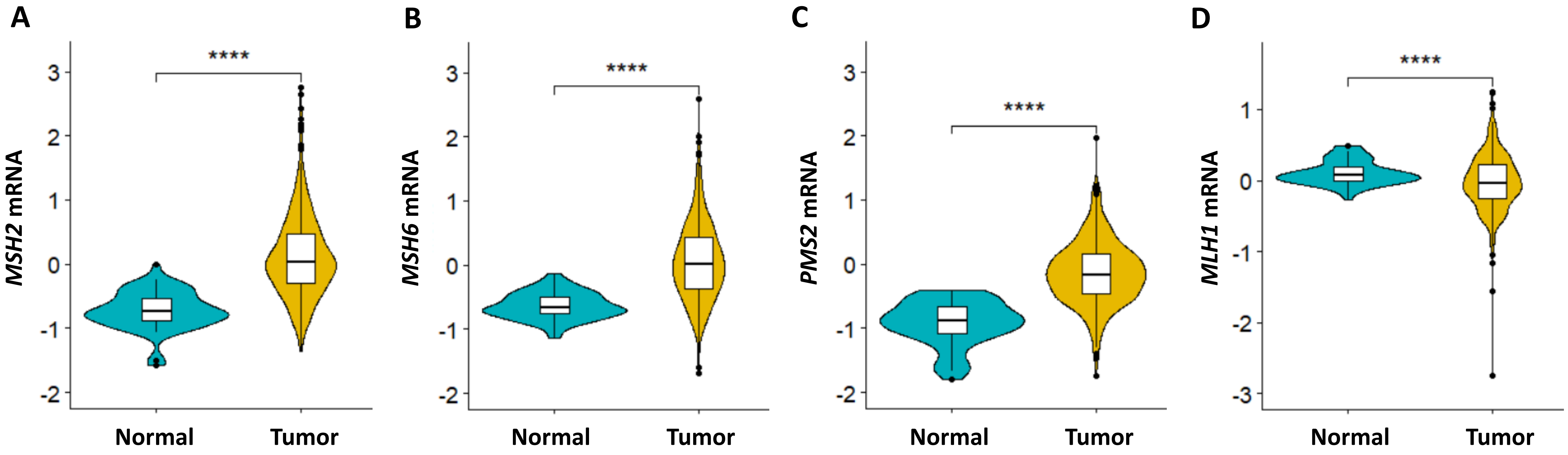

Figure S8. *MLH1* expression was specifically down-regulated in LUAD tissues. (A-D) Quantitative analysis of the mRNA expression of the four MMR genes in LUAD compared with adjacent normal tissues.
